# Supplementary material for: Pleural Effusions on MRI in Autosomal Dominant Polycystic Kidney Disease
Source: J Clin Med. 2023 Jan 3;12(1):386. doi: 10.3390/jcm12010386 (PMC9820892; doi:10.3390/jcm12010386)
Supplement: Supplementary file 1 [file jcm-12-00386-s001.zip › jcm-2085600-supplementary.pdf]

Supplemental Tables

**Supplemental Table S1.** Demographic and laboratory data in 110 ADPKD subjects, with and without pleural fluid. Normal distributed continuous variables are given as mean + standard deviation. Non-normal distributed continuous variables are given as mean (first quartile value - third quartile value). Ordinal variables are shown as number followed by the percentage.

| Demographic Data | No Pleural Fluid N=82 | Pleural Fluid N=28 | *p*-value |
|---|---|---|---|
| Age | 45±15 | 40±14 | 0.15 |
| Male: Female (% Male) | 44:38 (54%) | 8:20 (29%) | **0.02*** |
| *Race* | | | |
| White | 72 (87%) | 20 (71%) | |
| Black | 3 (4%) | 3 (11%) | **0.01*** |
| Asian | 4 (5%) | 3 (11%) | |
| Unknown | 3 (4%) | 2 (7%) | |
| Weight (kg) | 77 (62-88) | 72 (57-81) | 0.14 |
| Body Mass Index (kg/m2) | 26 (22 – 28) | 25 (21–27) | 0.54 |
| Body Surface Area (m2) | 1.9 (1.7-2.1) | 1.8 (1.6-2.0) | 0.08 |
| Systolic Blood Pressure (mmHg) | 122±14 | 122 (110-128) | 0.95 |
| Diastolic Blood Pressure (mmHg) | 78 (71-84) | 77 (72-82) | 0.79 |
| Estimated Glomerular Filtration Rate (ml/min/173m2) | 88 (67–109) | 93 (81–118) | 0.45 |
| Blood Urea Nitrogen (mg/dl) | 19 (14 – 21) | 17 (12–20) | 0.22 |
| Albumin (g/dl) | 4.4 (4.2 – 4.6) | 4.3 (4.2–4.4) | 0.12 |
| Aspartate Transaminase (U/L) | 27 (22 – 29) | 23 (17–27) | 0.10 |
| Alanine Transaminase (U/L) | 27 (17 – 29) | 21 (15–24) | 0.10 |
| Total Kidney Volume /height (ml/m) | 702 (333 – 959) | 719 (391–920) | 0.58 |
| Liver Volume (mL) | 1984 (1442–2113) | 1943 (1358–2044) | 0.84 |
| Spleen Volume (mL) | 255 (170 – 300) | 264 (207–308) | 0.70 |
| *Genotype Data* | | | |
| Data Available | 66 (80%) | 25 (93%) | 0.29 |
| PKD1 Mutations | 49 (74%) | 19 (76%) | 0.86 |
| PKD 2 Mutation | 17 (26%) | 6 (24%) | 0.86 |

* significant statistic difference exists ($p < 0.05$).
